# Classifiers of anterior cruciate ligament status in female and male adolescents using return‐to‐activity criteria

**DOI:** 10.1002/ksa.12462

**Published:** 2024-09-30

**Authors:** Céline I. Girard, Nicholas J. Romanchuk, Michael J. Del Bel, Sasha Carsen, Adrian D. C. Chan, Daniel L. Benoit

**Affiliations:** ^1^ Department of Mechanical Engineering University of Ottawa Ottawa Ontario Canada; ^2^ Ottawa‐Carleton Institute of Biomedical Engineering Ottawa Ontario Canada; ^3^ School of Rehabilitation Sciences University of Ottawa Ottawa Ontario Canada; ^4^ Division of Orthopaedic Surgery CHEO Ottawa Ontario Canada; ^5^ Department of Surgery University of Ottawa Ottawa Ontario Canada; ^6^ Department of Systems and Computer Engineering Carleton University Ottawa Ontario Canada; ^7^ School of Human Kinetics University of Ottawa Ottawa Ontario Canada; ^8^ Department of Health Sciences Faculty of Medicine Lund University Lund Sweden

**Keywords:** functional ability, machine learning, paediatric, sex difference, single limb hop, spatiotemporal measure

## Abstract

**Purpose:**

A lack of standardization exists for functional tasks in return‐to‐activity (RTA) guidelines for adolescents with anterior cruciate ligament injury (ACLi). Identifying the variables that discern ACLi status among adolescents is a first step in the creation of such guidelines following surgical reconstruction. This study investigated the use of classification models to discern ACLi status of adolescents with and without injury using spatiotemporal variables from functional tasks typically used in RTA guidelines for adults.

**Methods:**

Sixty‐four adolescents with ACLi and 70 uninjured adolescents completed single‐limb hops, lunges, squats, countermovement jumps and drop‐vertical jumps. Jumping distances, heights, and depths were collected. Decision trees (DTs) were used to classify ACLi status and were evaluated using the *F*‐measure (*F*_1_), kappa statistic (*ĸ*) and area under the precision–recall curve (PRC). Independent *t* tests and effect sizes were calculated for each important classifier of the DT models.

**Results:**

A five‐variable model classified ACLi status with an accuracy of 67.5% (*F*_1_ = 0.6842; *ĸ* = 0.350; PRC = 0.491) with sex as a classifier. Significant differences were found in three of the four spatiotemporal variables (*p* ≤ 0.002). Separate models then classified ACLi status in males and females with an accuracy of 53.3% (*F*_1_ = 0.5882; *ĸ* = 0.0541; PRC = 0.476) and 76.9% (*F*_1_ = 0.7692; *ĸ* = 0.541; PRC = 0.528), respectively, with significant differences for all variables (*p* ≤ 0.013).

**Conclusions:**

Among the DT models, females were better able to classify ACLi status compared to males, highlighting the importance of sex‐specific rehabilitation guidelines for adolescents.

**Level of Evidence:**

Level III.

**Abbreviations**
ACL: anterior cruciate ligament
ACLi: anterior cruciate ligament injury
ANT: maximal anterior hop
CMJ: countermovement jump
CON: uninjured control
CRS: cross hop for distance
DCrsDist: dominant/uninjured limb maximal perpendicular cross hop distance
DNormCrs: dominant/uninjured limb's cross hop distance normalized to body height
DT: decision tree
DTa: decision tree model differentiating adolescents with and without injuries
DTf: decision tree model differentiating injury status among females
DTm: decision tree model differentiating injury status among males
DVJ: drop vertical jump maximal lateral
F_1_
: *F*‐measure
LAT: maximal lateral hop
LOO: leave‐one‐out cross validation
LSI: limb symmetry index
LSLAT: limb symmetry index of the maximal lateral hop
LSTIME: limb symmetry index for the 6‐metre timed hop
LSTRP: limb symmetry index of the triple hop for distance
NNormANT: non‐dominant/injured limb ANT distance normalized to participants' heights
PRC: area under the precision–recall curve
PROM: patient‐reported outcome measure
RTA: return‐to‐activity
TIME: 6‐m timed hop
TRP: triple hop for distance
ĸ: kappa statistic

## INTRODUCTION

Annually, approximately 1 in 29 female athletes and 1 in 50 male athletes will experience an anterior cruciate ligament (ACL) injury [25, 43]. These injury rates have also been increasing by 2.3% every year for individuals between the ages of 10 and 18 [16, 68]. With these rising rates, particularly in females [25, 50, 68], there is a strong need to develop sex‐ and age‐specific evidence for the creation of return‐to‐activity (RTA) guidelines following an ACL injury in adolescent populations.

Adolescents' bodies are growing and changing throughout puberty. A knee injury in this key stage of their life, not only affects them physically but also socially because they may be side‐lined from their sports and activities with their friends for at least one year. Research in this area has increased to understand the injury mechanisms in adults [41, 46, 61] and create predictions for re‐injury risk within adolescents [51]. Sex differences have also been identified after the onset of puberty for neuromuscular [6, 22], biomechanical [19, 31] and clinical measures [15, 26]. However, the current guidelines for ACL injury (ACLi) and post‐operative rehabilitation do not account for sex differences in adults and have yet to be tested in adolescent males and females. Adolescents also reportedly have slower strength recovery following ACLi [33, 40], decreased neuromuscular control [53] and poor rates of passing RTA criteria [27, 48, 65]. With the greater risk of sustaining a secondary injury within two years of the initial ACLi [16, 29, 52, 67] and a 50% greater chance of developing early onset osteoarthritis within 5–20 years as compared to the general population [39, 45, 58], evidence‐based knowledge is needed to identify measures that can assess knee injury status following rehabilitation in adolescent patients with ACLi.

Clinicians use a range of functional capacity measures including questionnaires, strength tests, and functional tasks during the RTA assessment process to strengthen their clinical evaluations. Current functional tasks used in assessing rehabilitation progression and RTA include a series of single‐limb hopping tasks and other functional movements, such as lunges, squats, countermovement jumps and drop‐vertical jumps [4, 18, 70]; however, there is a lack of standardization for these functional tests in RTA assessments [57]. These tasks evaluate the control, strength, and power of the lower body limbs using a combination of raw scores, normalized scores, and limb symmetry indices to evaluate a patient's readiness to return to their pre‐injury level of physical activities. The recovered patient would receive a negative finding if features of ACLi are found in the patient following surgery and rehabilitation. To the best of our knowledge, there is no evidence identifying which spatiotemporal measures from functional tasks can differentiate uninjured and ACL‐injured adolescent males and females.

A classification model using a variety of functional tasks currently used in RTA evaluations may allow a tailored approach to be used in clinical settings to identify the effects of the ACLi on functional capacity in an adolescent population. At a minimum, the tasks and variables must identify the effect of the injury on functional capacity, so that the clinician can then assess if the deficits are recovered following surgery and rehabilitation. Such an algorithm could be used in conjunction with current clinical criteria to provide additional information for whether the patient is displaying functional capacity comparable to an uninjured cohort. Decision trees (DTs) are one of the most widely used techniques for classification [3, 60, 71] because of their simplicity [62], ease of implementation [3, 72], high interpretability and transparency and have been shown to provide a high accuracy across a variety of medical data sets [35, 38, 56]. This technique can be used to determine if a clinician should give consent for RTA based on the classification of ACLi status using objective measures since a DT is like a flow chart, where each node denotes a test on an attribute, each branch represents the outcome of the test, and each leaf represents a class [38]. Therefore, the purpose of this study was to (1) identify tasks and variables capable of classifying ACLi status in adolescents and (2) identify unique tasks for ACLi status classification in both male and female adolescents. We hypothesized that the classifiers extracted would differ between models for male and female adolescents.

## MATERIALS AND METHODS

Sixty‐four (45 female, 19 male) adolescents with an ACL injury (ACLi; 15.3 ± 1.3 years; 167.7 ± 8.1 cm; 68.1 ± 19.6 kg; BMI: 24.1 ± 6.0 kg/m^2^; Tanner Stage: 4 ± 1) and 70 (39 female, 31 male) uninjured (CON; 13.8 ± 1.7 years; 165.0 ± 10.8 cm; 54.0 ± 11.8 kg; BMI: 19.6 ± 2.9 kg/m^2^; Tanner Stage: 4 ± 1) adolescents participated in this study (Table 1). ACLi participants were included in this study if they had a confirmed ACL rupture by their physician via clinical assessment and/or magnetic resonance imaging and subsequent arthroscopy, and no history of contralateral ACL ruptures, excluding those seeking an ACL reconstruction revision. The CON participants were recruited via convenience and snow‐ball sampling of regional youth sports associations and club teams. The CON participants and the contralateral limb of the ACLi group were also required to not have any previous lower limb surgical operations, no additional serious lower limb injury including fractures or sprains within 6 months prior to testing or have any diseases that might affect neuromuscular function. Each CON participant actively participated in organized sports at the time of testing, as assessed through the HSS Pedi‐FABS or FR Pedi‐FABS [17, 21]. Pre‐injury and current physical activity levels as defined by the Tegner Activity Level Scale were included for all participants [64].

**Table 1:** Participant characteristics and subjective functional scores.

Variable	Uninjured male (*n* = 31)	Uninjured female (*n* = 39)	ACL‐injured male (*n* = 19)	ACL‐injured female (*n* = 45)
	(a) Mean ± SD	(a) Mean ± SD	(a) Mean ± SD	(a) Mean ± SD
	(b) Median (range)	(b) Median (range)	(b) Median (range)	(b) Median (range)
Age (years)	(a) 13.7 ± 1.7*	(a) 13.9 ± 1.8^§^	(a) 15.4 ± 1.2*	(a) 15.3 ± 1.3^§^
Height (m)	(a) 1.67 ± 0.01	(a) 1.63 ± 0.07	(a) 1.74 ± 0.09	(a) 1.65 ± 0.06
Weight (kg)	(a) 54.4 ± 12.6*	(a) 53.7 ± 11.3^§^	(a) 76.3 ± 31.0*	(a) 64.7 ± 10.9^§^
Time since injury (months)	‐	‐	(a) 5.6 ± 2.9	(a) 5.5 ± 4.2
HSS/FR Pedi‐FABS (0–30)	(a) 26 ± 4*	(a) 21 ± 5^§^	(a) 9 ± 8*	(a) 7 ± 6^§^
Pre‐injury Tegner Activity Scale (0–10)	‐	‐	(b) 9 (10–6)	(b) 9 (10‐6)
Current Tegner Activity Scale (0–10)	(b) 9 (10–5)*	(b) 9 (9–5)^§^	(b) 5 (9–0)*	(b) 3 (9–0)^§^
Tanner Stage (1–5)	(a) 4 ± 1	(a) 4 ± 1	(a) 4 ± 1	(a) 4 ± 1

*Note*: Statistically significant differences (*p* < 0.05) between male and female (* and ^§^, respectively) CON versus ACLi are identified.

Abbreviations: ACLi, anterior cruciate ligament injury; CON, uninjured control.

During the study, participants wore tight‐fitting spandex shorts and a long‐sleeved shirt. Anthropometric measurements were taken, including height (cm) and weight (kg). Participants were outfitted with 84 reflective markers (14 mm diameter) placed on various landmarks according to a hybrid marker‐cluster set [5]. Participants were then given the opportunity to stretch and spin on a cycle ergometer (Monark 828En) with minimal resistance for 5 min as a warm‐up.

Each participant was asked to complete a series of dynamic movement tasks, including a battery of single‐limb hops [24, 44], lunges [1], bilateral squats [66], countermovement jumps (CMJ) [30] and drop vertical jumps (DVJ) [47]. The distance between the starting position indicated by a zero on the measurement tape and the heel marker's landing position of the test limb was used to quantify hopping distance for the maximal anterior (ANT), maximal lateral (LAT), triple (TRP) and cross (CRS) hops. In addition, the maximal lateral distance between the three hops for the CRS and the time to complete the 6‐m timed (TIME) hop were measured totalling 12 variables for the single‐limb hopping tasks. Two good trials were recorded for each hopping task and the best score (i.e., greatest distance or shortest time) was used in the model. These tasks and variables were chosen because they could all be measured in a clinical setting with a stopwatch and a measuring tape [23].

Three‐dimensional motion capture data were recorded (8 Vero, 2 Vantage, Vicon), sampling at 200 Hz to extract the spatiotemporal variables from the other functional tasks including the lunges, squats, CMJ and DVJ. The lunge and squatting depth were measured for the lunges and bilateral squats, respectively, based on the vertical difference in the pelvis origin. Similarly, the pelvic origin was used to compute the squatting depth and jump height for the CMJ. A good trial for the lunge, squat and CMJ was defined by keeping their hands on their head and maintaining their balance throughout the trial. The squatting depth at both the initial takeoff after stepping off the platform onto the ground and the final landing phase, and the jump height relative to their standing static trial was used for the DVJ. A good DVJ trial consisted of participants stepping off the platform, not jumping off and maintaining their balance for two seconds after the final landing. Five good trials were recorded for the additional functional tasks and the best score (i.e., greatest depth or jump height) was used as the input variable in the model.

The number of participants contributing to each task varied as trials were deemed missing variables in the analysis where participants either did not perform a particular movement due to their comfort level or where missing data did not permit a complete marker reconstruction for the duration of the task (Table 2). Distances, depths and jump heights were normalized to the participant's height and both raw and normalized variables were included in the DT model for all contributing participants. For all six distance‐based jumping tasks and lunges performed on each limb separately, limb symmetry indices (LSIs) were calculated using the following equation: LSI = Injured/Uninjured or Non‐dominant/Dominant. These nine tasks and 45 variables were chosen because they could all be measured in a clinical setting with a stopwatch and a measuring tape to represent clinically available measures.

**Table 2:** Number of ACL‐injured participants contributing to each task.

Task	Maximal anterior hop	Maximal lateral hop	Triple hop	Cross hop	6‐metre timed hop	Lunges	Squats	Countermovement jump	Drop vertical jump
Male (*n* = 19)	18	16	14	13	16	18	19	19	18
Female (*n* = 45)	40	38	39	31	38	45	45	43	40

Abbreviation: ACL, anterior cruciate ligament.

## STATISTICAL ANALYSIS

The C5.0 DT model was used, the successor to C4.5 and ID3, as its memory usage is more efficient and produces smaller DTs with lower error rates on testing sets [49]. The C5.0 algorithm provides feature selection, cross‐validation and reduced error pruning [7, 9]. The algorithm treats missing values by redistributing the weights of the variable branch for which the value is missing using the C4.5 algorithm [55]. In this case, the C5.0 algorithm was set at seed 4, with the minimum number of cases used in at least two of the splits as the length of the training set divided by the number of splits used in each iteration of the cross‐validation minus 1 [9, 54], with global pruning enabled, and leave‐one‐out‐cross‐validation (LOO).

The LOO was applied to 70% of the data, with the remaining 30% of the data used as the test set for performance analysis. LOO uses one observation (i.e., one participant) as the testing set and builds the model on the remaining *n* − 1 training participants and repeated *n* times so that each participant is used as the test set to tune the hyperparameters and validate the model simultaneously [13]. The LOO estimate is then the average of these *n* test error estimates. This process allows for all participants to be used in both training and validation to identify optimal hyperparameters for the DT model and can then be tested to evaluate its generalizability with unseen data.

**Figure 1:**
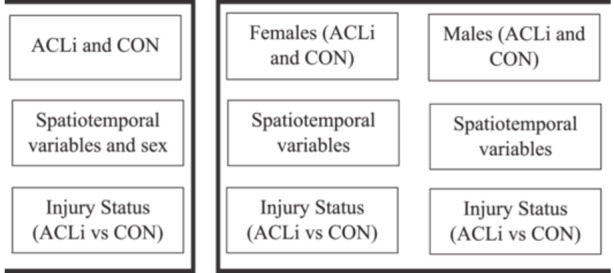
Review of data breakdown for each model with the group, inputted features and differentiating classes outlined. ACLi, anterior cruciate ligament injury; CON, uninjured control.

A twofold approach was used to assess injury status and sex differences. A DT model was first created to differentiate adolescents with and without injuries (DTa). All variables, including sex, were used as features in the DTa model to explore their importance in the separation of ACLi status (Figure 1). Separate models were then created for each sex to explore the functional tasks chosen as classifiers for differentiating injury status among females (DTf) and males (DTm) (Figure 1).

The data for DTa were first randomized and split into training and testing sets with classes and sex equally distributed between sets for a 70/30 split. For DTf and DTm, the data were randomized with classes equally distributed between sets for the 70/30 split. A class weight was then assigned to account for imbalanced classes using the following equation: *w*_*j*_ = *N*_*T*_/(*C*_*j*_ * *N*_*j*_), where *w*_*j*_ is the weight for class *j*, *N*_*T*_ is the total number of participants in the data set, *C*_*j*_ is the total number of unique classes (2) and $N_{j}$ is the total number of participants within the respective class.

The performance of each classifier was evaluated on the test set (30% of the data) using the *F*‐measure (*F*_1_), kappa statistic (*ĸ*) and the area under the precision–recall curve (PRC). The *F*_1_ represents the harmonic mean between precision and recall [32], and the *ĸ* was used to compare the observed accuracy with the expected accuracy and takes into account random chance [12]. The PRC describes how good a model is at predicting the positive class [34] and can reveal differences in performance when using imbalanced data sets [59]. The confusion matrix for the models was constructed, and the sensitivity and specificity outlined to represent clinically relevant performance measures [28].

Independent *t* tests (or the non‐parametric equivalent, Mann–Whitney *U* tests) and effect sizes were calculated for each important classifier determined through the DT model. Small effect size were considered to be *d* < 0.3, medium 0.3 < *d* < 0.5, and large *d* > 0.5 [14]. All statistical analyses were performed using R (RStudio) with a significance set at *α* = 0.05.

## RESULTS

**Figure 2:**
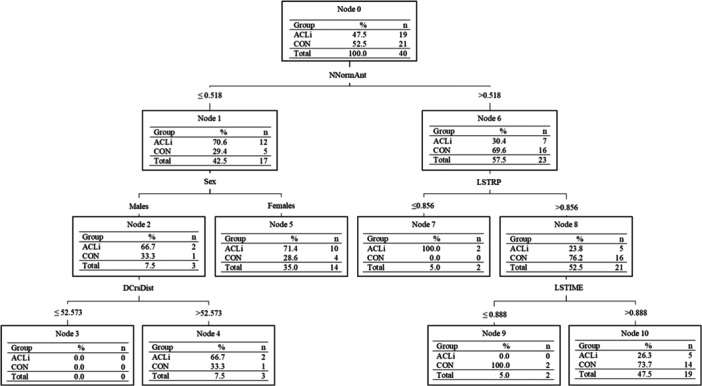
C5.0 decision tree model for both male and female adolescents in the testing set differentiating those with anterior cruciate ligament injury (ACLi) participants and uninjured control (CON). DCrsDist, dominant/uninjured limb's maximal perpendicular cross‐hop distance; LSTIME, limb symmetry index of the 6‐m timed hop; LSTRP, limb symmetry index of the triple hop distance; NNormAnt, nondominant/injured limb's maximal anterior hop normalized to body height.

To differentiate ACLi status for the 94 participants in the training set, 47 variables were reduced to a five‐variable model (DTa) including nondominant/injured limb ANT distance normalized to participants' heights (NNormANT), LSI of the TRP for distance (LSTRP), limb symmetry index for the TIME hop (LSTIME), sex and dominant/uninjured limb maximal perpendicular CRS distance (DCrsDist) (Figure 2). Significant differences were identified for NNormANT (*p* < 0.001; *d* = 0.838), LSTRP (*p* < 0.001; *d* = 0.789) and LSTIME (*p* = 0.002; *d* = 0.557). The variable DCrsDist was not statistically different between groups.

**Figure 3:**
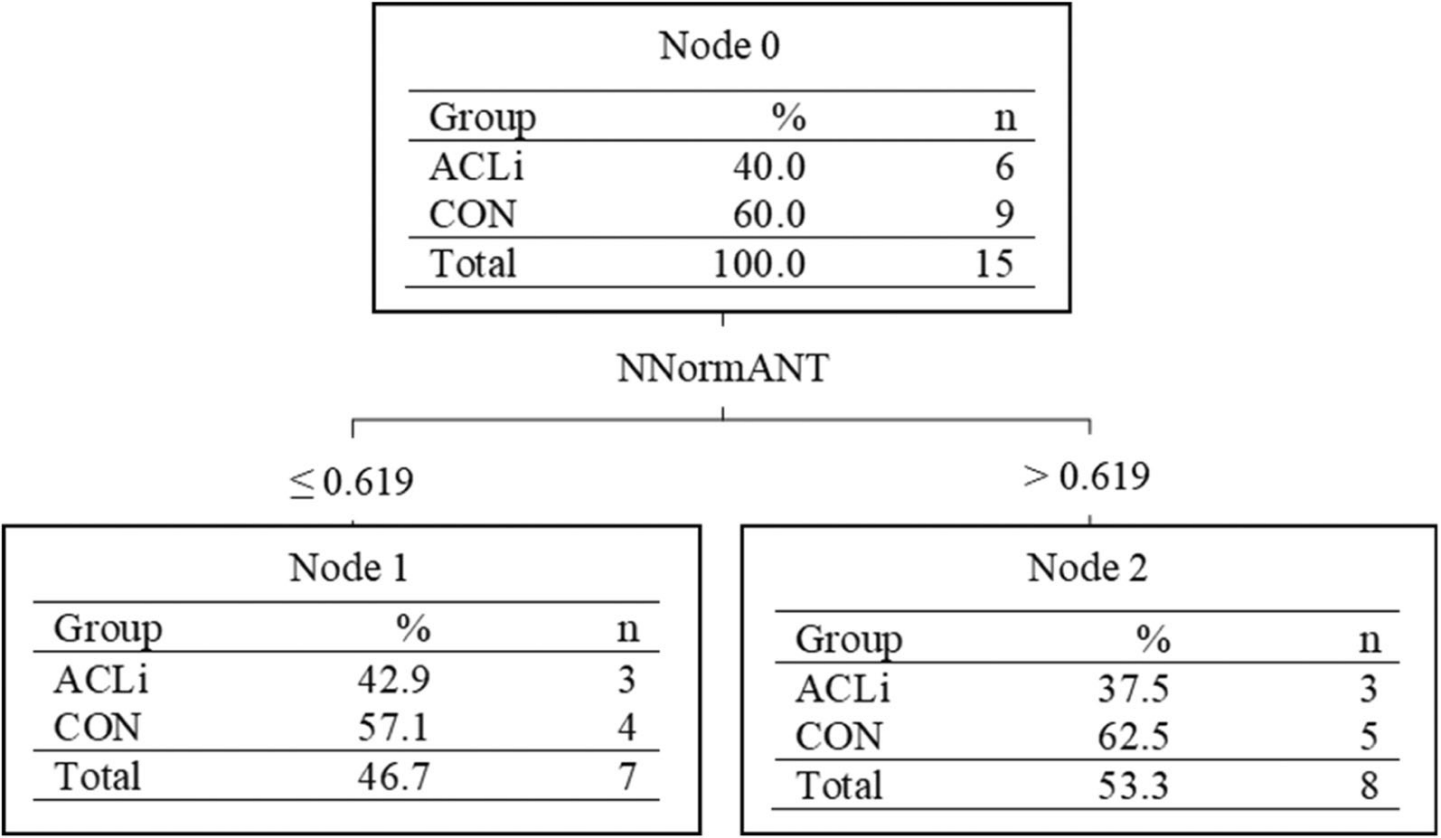
C5.0 decision tree model for male adolescents in the testing set differentiating those with anterior cruciate ligament injury (ACLi) and uninjured control (CON). NNormAnt, non‐dominant/injured limb's maximal anterior hop normalized to body height.

The DT model for ACLi status prediction in the male adolescents (DTm) highlighted the NNormANT as the most important classifier (*p* = 0.013; *d* = 0.783) (Figure 3).

**Figure 4:**
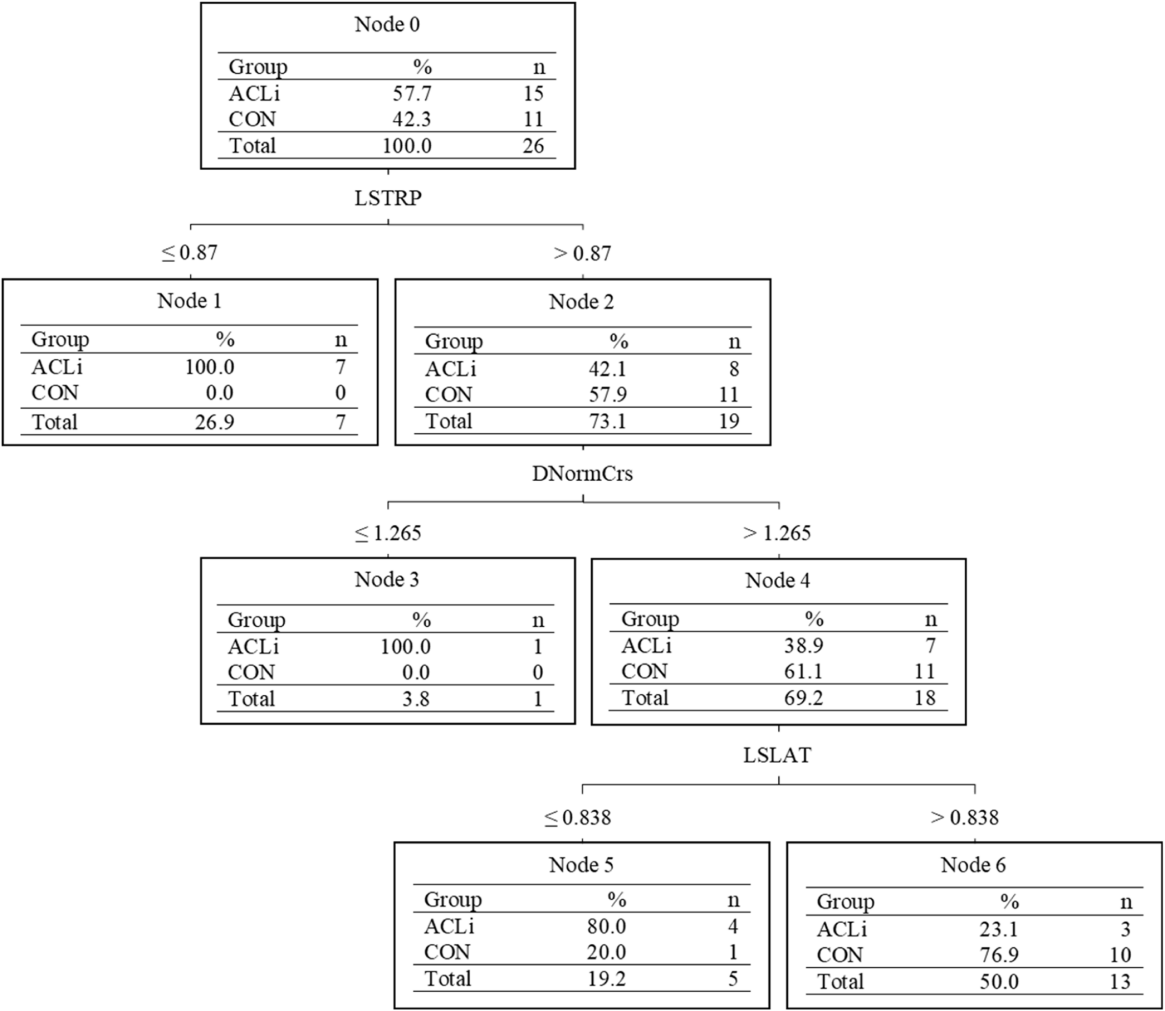
C5.0 decision tree model for female adolescents in the testing set differentiating those with anterior cruciate ligament injury (ACLi) and uninjured control (CON). DNormCrs, dominant/uninjured limb's maximal cross hop distance normalized to body height; LSLAT, limb symmetry index of the maximal lateral hop distance; LSTRP, limb symmetry index of the triple hop distance.

The LSTRP was identified as the most important classifier in the DT model for ACLi status prediction in the female adolescent group (DTf; *p* < 0.001; *d* = 1.156). After LSTRP, the dominant/uninjured limb's cross hop distance normalized to body height (DNormCrs) was identified as an additional classifier improving the model's predictability (*p* = 0.012; *d* = 0.565), with the LSI of the LAT (LSLAT) as the final classifier in the model (*p* < 0.001; *d* = 0.749) (Figure 4). Table 3 compares the descriptive results of the identified classifiers' scores between groups, and Table 4 outlines the results of each of the three models studied using the reduced variables on the test set. The female group outperformed males and the entire group's DT models on all performance metrics when differentiating ACLi status.

**Table 3:** Descriptive statistics of the raw scores for the classifier variables chosen within the female and male C5.0 models.

		Males	Females		
Measure		NNormAnt	LSTRP	DNormCrs	LSLAT
Mean ± SD	ACLi	0.478 ± 0.166	0.870 ± 0.126	1.598 ± 0.394	0.856 ± 0.243
	CON	0.635 ± 0.166	0.990 ± 0.077	1.801 ± 0.346	1.001 ± 0.124
Range	ACLi	0.140–0.694	0.528–1.345	0.591–2.731	0.328–1.805
	CON	0.273–1.065	0.855–1.234	1.134–2.412	0.646–1.322

Abbreviations: ACLi, anterior cruciate ligament injury; CON, uninjured control; DNormCrs, dominant/uninjured limb's maximal cross hop distance normalized to body height; LSLAT, limb symmetry index of the maximal lateral hop distance; LSTRP, limb symmetry index of the triple hop distance.

**Table 4:** Evaluation metrics of the testing sets on the C5.0 models.

Measure	Entire group (both males and females)	Males	Females
Accuracy	67.5%	53.3%	76.9%
Sensitivity	70.0%	62.5%	71.4%
Specificity	65.0%	42.9%	83.3%
*F*‐measure	0.6842	0.5882	0.7692
Kappa statistics	0.350	0.0541	0.541
PRC area	0.491	0.476	0.528

Abbreviation: PRC area under the precision–recall curve.

## DISCUSSION

The purpose of this study was to examine if spatiotemporal variables extracted from functional tasks typically used in RTA assessments could differentiate ACLi status in adolescent males and females. The model differentiating the female adolescent groups correctly classified 76.9% of participants with three classifiers compared to 53.3% with one classifier and 67.5% with four classifiers for the males and the entire group, respectively. The results suggest that a total of six variables (excluding sex) within the battery of five single‐limb hopping tasks [24, 44] can differentiate ACLi status in adolescents. The male group demonstrated a classification ability slightly greater than chance (>50%); however, had no agreement with the group truth (*ĸ* = 0.0541), whereas the female group offered an overall better classification performance. Differences in performance metrics between sexes were largely governed by the different tasks chosen by the C5.0 DT algorithm for the classification of ACLi status. Furthermore, the addition of sex as a classifier within the model that included both males and females (Figure 2) further justifies separating sexes when evaluating their functional performance before and after injury to a lower limb.

After the onset of puberty, differences in males and females begin to emerge with respect to their anatomy and muscular composition. This can lead to differences in performance metrics which is why many studies normalize strength and functional task variables to either body weight or height [37]. We used a combination of raw and normalized scores for each task to evaluate their ability to differentiate the adolescent groups. Interestingly, limb symmetry indices and normalized values were used in the models despite no significant height differences between the injured and uninjured groups within each sex (Table 1). Although the injured participants were slightly older and therefore heavier than their uninjured counterparts, the LSIs observed in the younger uninjured group reflect those of an adult population [36, 42]. Furthermore, there were no group differences between the raw scores of the dominant limb for the CON group and the uninjured limb for the ACLi group in the single‐limb hopping scores of the maximal anterior hop, maximal lateral hop, cross hop and triple hop in both the male and female groups. These raw scores were similar to a previous study that examined the single‐limb hopping scores in an adolescent population in both a laboratory setting and a physical education class [23]. This suggests that the age difference between our injured and uninjured cohorts would not have biased these findings. That being said, the female group remained easier to separate with larger ranges in the injured group compared to the uninjured group (Table 3). For example, the range for the injured group's LSLAT classifier is 0.328–1.805 which indicates highly asymmetrical values with either the injured or uninjured limbs having different performances with respect to the other limb. Furthermore, the female group's model used the DNormCrs classifier which uses the dominant limb of the control group or the uninjured limb of the group with ACLi. Previous studies have reported worse self‐reported outcomes for females than males [20, 63] with longer recovery times and strength recovery post‐reconstruction [27, 37]. The decrease in physical activities compared to the male group (Table 1) may begin to explain the large differences between the two sexes, in addition to the deconditioning of the uninjured limb after injury. This suggests the importance for both males and females to remain physically active prior to their surgery. This further highlights the importance of sex‐specific rehabilitation guidelines and monitoring to ensure female athletes are returning to their sport with confidence, adequate muscular strength and in a timely manner.

Most agree on using a battery of tests to assess RTA readiness, including muscle strength testing, functional tests, movement quality assessments, and patient‐reported outcome measures (PROMs) [10, 11]. It can be difficult to decipher which tests to use especially when time and space are limited. Using a classification model that provides a logical order of tests and measures to use may aid in the decision‐making process to determine readiness to return to pre‐injury activities following reconstruction. Such a classification method does not, however, come without its limitations and still requires some knowledge and expertise with respect to the decisions taken in the model.

The current C5.0 DT models that classified ACLi by sex in the test set misclassified six females (four ACLi; two CON) and seven male participants (three ACLi; four CON). One of the female ACLi participants had not completed any of the hop tasks because of their discomfort level. It can therefore be assumed this patient does not have the functional capacity required to be labelled as an uninjured adolescent; and should not have been labelled as such. If done manually, as is the case with Figures 2, 3, 4, the participant would be correctly classified as they did not achieve values greater than the thresholds of each node. The correct classification of ACLi patients becomes more difficult when these patients have functional performances similar to their uninjured cohort. This was the case for six ACLi participants, whose performances fell within 1 SD of the mean of the uninjured group and were mislabelled as uninjured. These ACLi participants may fall within a subgroup deemed copers who would be able to return to the same activity level as before their injury [2]; however, a deeper analysis is warranted to confirm whether they fall within this subgroup. In addition, traditionally, statistical tests have been geared towards the testing of a single hypothesis, whereas data mining through machine learning classifiers focuses on the search through multiple potential hypotheses [8, 69]. This approach allows for the testing and tailoring of multiple clinical tests for ACLi status classification and thereby may provide clinicians with a set of tests for rehabilitation/RTA assessments following surgical reconstruction. Although a great first step in the tailoring of functional tasks in an adolescent population, additional measures such as their muscular strength and PROMs should be used in conjunction to functional tasks during a patient's rehabilitation and RTA assessment to minimize misclassification of the patient group.

In this work, we have explored the use of an algorithm to discern knee injury status using clinically accessible measures across a variety of functional tasks currently used in RTA evaluations. The battery of single‐limb hopping tasks classified participants with and without ACLi, with the maximal anterior hop classifying ACLi status in males, and the triple hop, maximal lateral hop, and cross hop classifying ACLi status in female adolescents. The C5.0 DT model was better able to classify females compared to their male counterparts highlighting the importance of sex‐specific rehabilitation guidelines. This technique may be used to determine if a clinician should give consent for RTA based on the classification of ACL injury status using objective measures with a flow‐chart like decision‐based algorithm [38]. Furthermore, the tasks and variables chosen as classifiers in each model can be easily completed in a clinical setting and may be utilized in conjunction with other current measures to help clinicians determine if patients are exhibiting performances similar to their uninjured counterparts at the time of an RTA decision.

## CONCLUSION

Among the evaluated models, DTf had the highest classification performance for differentiating ACLi status in adolescents. Female adolescents were classified with 76.9% accuracy with three spatiotemporal variables including the limb symmetry of the triple hop, the dominant/uninjured limb's normalized cross hop score, and the limb symmetry of the lateral hop. However, the inability to classify ACLi status among the male group with confidence suggests that these spatiotemporal measures are inadequate on their own for evaluating RTA readiness following surgical reconstruction. Therefore, future rehabilitation guidelines for adolescents should consider evaluating females and males separately to determine readiness to RTA using the cut‐offs provided in this study as baseline ACLi status function.

## AUTHOR CONTRIBUTIONS

All authors contributed to the study's conception and design. Céline I. Girard, Nicholas J. Romanchuk and Michael J. Del Bel prepared the material and performed the data collection and analysis. The first draft of the manuscript was written by Céline I. Girard and all authors commented on previous versions of the manuscript. All authors have read and approved the final manuscript.

## CONFLICT OF INTEREST STATEMENT

The authors declare no conflict of interest.

## ETHICS STATEMENT

All participants provided written informed consent approved by the hospital and institutional ethics review boards and were part of a larger ongoing prospective, repeated measures cohort study.

## Data Availability

The data that support the findings of this study are available from the corresponding author upon reasonable request.
